# Health‐related needs reported by adolescents living with HIV and receiving antiretroviral therapy in sub‐Saharan Africa: a systematic literature review

**DOI:** 10.1002/jia2.25921

**Published:** 2022-08-19

**Authors:** Elvis D. Chem, Alissa Ferry, Janet Seeley, Helen A. Weiss, Victoria Simms

**Affiliations:** ^1^ Department of Infectious Disease Epidemiology London School of Hygiene & Tropical Medicine London UK; ^2^ Plan International Germany Hamburg Germany; ^3^ Department of Global Health and Development London School of Hygiene & Tropical Medicine London UK

**Keywords:** adolescents, antiretroviral therapy, ART adherence, health needs, HIV

## Abstract

**Introduction:**

Adolescents living with HIV (ALHIV) on antiretroviral therapy (ART) have specific health needs that can be challenging to deliver. Sub‐Saharan Africa (SSA) is home to 84% of the global population of ALHIV, of whom about 59% receive ART. Several studies in SSA have demonstrated health service gaps due to lack of synchronized healthcare for ALHIV receiving ART. We conducted a systematic review of health‐related needs among ALHIV on ART in SSA to inform decisions and policies on care.

**Methods:**

We searched MEDLINE, Web of Science, EMBASE, PsycINFO, Cochrane library and grey literature for studies reporting health‐related needs among ALHIV receiving ART in SSA, between January 2003 and May 2020.

**Results and discussion:**

Of the 2333 potentially eligible articles identified, 32 were eligible. Eligible studies were published between 2008 and 2019, in 11 countries: Zambia (7), Uganda (6), Tanzania (4), South Africa (4), Kenya (3), Ghana (2), Zimbabwe (2), Rwanda (1), Malawi (1), Botswana (1) and Democratic Republic of Congo (1). Seven categories of health needs among ALHIV were identified. In descending order of occurrence, these were: psychosocial needs (stigma reduction, disclosure and privacy support, and difficulty accepting diagnosis); dependency of care (need for family and provider support, and desire for autonomy); self‐management needs (desire for better coping strategies, medication adherence support and reduced ART side effects); non‐responsive health services (non‐adolescent friendly facility services and non‐compatible school system); need for food, financial and material support; inadequate information about HIV (desire for more knowledge to fight misinformation and misconception); and developmental and growth needs (desire to experience sex, parenthood and love). Ecological analysis identified different priority needs between ALHIV, their caregivers and healthcare providers, including psychosocial needs, financial challenges and non‐responsive health services, respectively.

**Conclusions:**

To respond effectively to the health needs of ALHIV and improve ART adherence, interventions should focus on stigma reduction, disclosure challenges and innovative coping mechanisms for ART. Interventions that address the health needs of ALHIV from the perspective of carers and providers, such as financial support schemes and adolescent‐friendly healthcare strategies, should supplement efforts to improve adolescent ART adherence outcomes.

## 1 INTRODUCTION

Adolescents living with HIV (ALHIV) receiving antiretroviral therapy (ART) have specific health needs that can be challenging to deliver compared with adults. Worldwide, an estimated 1.8 million adolescents (10–19 years) are living with HIV, of whom about 89% reside in sub‐Saharan Africa (SSA) [1, 2]. In the last decade, there has been clinical progress in the management of HIV among ALHIV with improvements in survival rates and increased life expectancy [3], and since 2015, the number of ALHIV has begun to decline [UNAIDS 2021]. This is partly due to the benefits of ART, increased uptake of the prevention of mother to child program, which has reduced new infections in children, and increased survival of ALHIV into adulthood. Despite these gains, incidence rates of new HIV infections as well as HIV‐related mortality remain high among ALHIV when compared to other age groups [2]. The majority of ALHIV aged 15–19 years in SSA are females (61%), and two‐thirds of new infections are among females [4, 5].

Achieving and sustaining the transformative effects of ART among ALHIV depends on consistent access and good adherence to ART. While access to ART among ALHIV is increasing in low‐ and middle‐income countries (LMICs) [6], adherence remains challenging. There are multiple reasons for this, as adolescence is characterized by significant cognitive, physical, emotional and incomplete psychological development, which affects adolescent's perceptions, decision making and actions, such as engagement with healthcare services. These adolescent development challenges are exacerbated for those living with HIV who require long‐term ART adherence to achieve good health outcomes [3]. Adherence barriers faced by ALHIV include social barriers, such as HIV‐related stigma, and structural factors, including poverty, food insecurity, violence victimization and overcrowded households with little privacy. Hence, ALHIV are often financially dependent and lack autonomy, rely on support from their caregivers and healthcare personnel to sustain ART adherence and to develop agency over ART medication [7, 8]. Once diagnosed and initiated onto ART, ALHIV in LMICs experience high rates of loss to follow‐up due to lack of organized systems of health services, such as consistent availability of ART; adequate human, material and infrastructural resources; appropriate ART adherence follow‐up plan; and psychosocial support mechanisms [3].

There is an evidence gap regarding the unmet health needs of ALHIV receiving ART in SSA. Limited funding and lack of resources for effective HIV services have weakened efforts to improve adherence to ART among ALHIV in SSA emphasizing the need to optimize available resources by investigating the health‐related needs among ALHIV to focus service delivery efforts on priority areas with the greatest impact for ALHIV in SSA [9].

The second UNAIDS fast track target for 2030 aims to achieve 95% ART initiation among all ALHIV. Among the estimated 1.8 million ALHIV globally, 59% were receiving ART as of 2020 in UNAIDS focus countries in SSA [10]. Despite the multitude of studies that have investigated the needs and experiences of ALHIV on ART in different settings within SSA [11, 12, 13], no systematic review has been conducted to pull together the evidence to inform policies around priority interventions that can improve ART services and adherence outcomes for ALHIV in SSA. A review of health service gaps for ALHIV receiving ART in LMICs identified gaps in care but did not take a need‐based approach [14]. To address this evidence gap, we conducted a systematic review of health‐related needs experienced by ALHIV on ART in SSA, to highlight areas for policy improvement, further research and facilitate the choice and development of targeted interventions that address the health needs of ALHIV on ART [9, 15].

This review aims to summarize the evidence on the felt and expressed health‐related needs of ALHIV and those closely involved in their care [16, 17], including their need for, and use of health services [18]. In line with the health systems approach, the WHO definition has been adopted, which states that health needs are “*objectively determined deficiencies in health that require health care, from promotion to palliation.”* [19].

## 2 METHODS

The protocol for this review was registered at the International Prospective Register of Systematic Reviews (ID CRD42019160425) and followed the Preferred Reporting Items for Systematic Reviews and Meta‐analysis (PRISMA) guidelines [20].

### 2.1 Search strategy

In May 2020, we searched five databases: MEDLINE, Web of Science, EMBASE, PsycINFO and Cochrane library for studies reporting health‐related needs among ALHIV on ART in SSA. The International AIDS conference database was searched between January 2003 and May 2020. Manual search for additional articles from the bibliographies of relevant articles and systematic reviews was conducted iteratively. Grey literature was searched using Google Scholar, USAID's AIDS Free Project and the PEPFAR websites.

The search strategy was designed and modified across different databases (File S1). Primary searches were conducted without restrictions on language or publication status (peer‐reviewed publication, unpublished, reports and press articles). However, following primary search, only publications in English were considered.

### 2.2 Inclusion criteria

Studies were eligible if they (1) investigated the self‐reported needs of ALHIV on ART; (2) investigated healthcare providers or caregiver's perspective of health needs for ALHIV on ART; (3) were conducted in SSA; and (4) were published between January 2003 and May 2020.

### 2.3 Study selection and data abstraction

Retrieved studies were exported and saved in EndNote X9, and deduplicated. For studies whose abstracts were retrieved through database search and had no full text, the authors of the papers were contacted to request the full text. Screening of titles and abstracts was conducted by two screeners (EDC and AF), and non‐eligible records were excluded. Discrepancies between the screeners were resolved through consensus. Post‐screening, the full text of retained studies was read to further assess eligibility. Articles were excluded at this stage with a reason, and the final list of records for data extraction was developed.

A data abstraction form was developed in Microsoft Excel, pilot tested on five studies, modified and used for abstraction of data from all included studies. The PDF versions of the retained studies were later imported into NViVo 12, read and coded. Codes obtained through NViVo were compared to those generated manually from the excel data. The descriptive characteristics of the studies abstracted included author, study year, study design, country, sample size, adolescent age, study participants and mode of HIV acquisition (Table 1).

**Table 1:** Eligible studies retained in the review

Author, year	Study design	Country	Study participants and sample size	Mode of acquisition	ALHIV age range
Ferrand, et al. [22]	Cross‐sectional survey	Zimbabwe	HCP and HIV program managers (115 facilities)	NR	10–19
Hodgson, et al. [11]	Qualitative cross sectional	Zambia	ALHIV (111), caregivers (21) and HCP (38)	NR	10–19
Denison, et al. [23]	Qualitative cross sectional	Zambia	ALHIV (32) and caregivers (23)	Perinatal	15–18
Mburu, et al. [12]	Qualitative cross sectional	Zambia	ALHIV (111), caregivers (21) and HCP (38)	Perinatal and horizontal	10–19
Bakeera‐Kitaka, et al. [24]	Qualitative cross sectional	Uganda	ALHIV (75) and HCP (12)	NR	11–21
Busza, et al. [13]	Qualitative cross sectional	Tanzania	ALHIV (14), caregivers (10) and HCP (12)	NR	15–24
Abubakar, et al. [25]	Qualitative cross sectional	Kenya	ALHIV (12), HIV‐negative adolescents (12), caregivers (11), HCP (8) and teachers (6)	NR	12–17
Enimil, et al. [26]	Mixed methods	Ghana	ALHIV (60)	Perinatal	12–19
Ankrah, et al. [27]	Qualitative cross sectional	Ghana	ALHIV (19)	Perinatal and behavioural	12–19
Dow, et al. [28]	Cross sectional survey	Tanzania	ALHIV (182)	NR	12–24
Birungi, et al. [29]	Mixed methods	Uganda	ALHIV (48)	Perinatal	15–19
Birungi, et al. [30]	Mixed methods	Uganda	ALHIV (48) and HCP (4)	Perinatal	15–19
Hagey, et al. [31]	Qualitative cross sectional	Kenya	HCP (39)	NR	
Mavhu, et al. [32]	Mixed methods	Zimbabwe	ALHIV (56), caregivers and HCP (72)	NR	15–18
Luseno, et al. [33]	Qualitative cross sectional	Kenya	ALHIV (29) and caregivers (14)	Perinatal and behavioural	15–19
Li, et al. [34]	Qualitative cross sectional	South Africa	ALHIV (26)	NR	7–15
Fetzer, et al. [35]	Qualitative cross sectional	Democratic Rep. of Congo	ALHIV (20) and caregivers (20)	NR	8–17
Rutakumwa, et al. [36]	Qualitative longitudinal	Uganda	ALHIV (40) and caregivers (40)	NR	13–17
Kajubi, et al. [37]	Qualitative cross sectional	Uganda	ALHIV (29) and caregivers (29)	Perinatal	8–17
Vujovic, et al. [38]	Qualitative cross sectional	South Africa	ALHIV (27) and caregivers (9)	Perinatal	10–14
Okawa, et al. [39]	Cross sectional survey	Zambia	ALHIV (175)	NR	15–19
McCarraher, et al. [40]	Mixed methods	Zambia	ALHIV (32), caregivers (23) and HCP (10)	Perinatal and behavioural	15–18
Mwalabu, et al. [41]	Qualitative case study approach	Malawi	ALHIV (14), caregivers (14) and HCP (14)	NR	15–19
Okawa, et al. [42]	Mixed methods	Zambia	ALHIV (200)	NR	15–19
Mutwa, et al. [43]	Qualitative cross sectional	Rwanda	ALHIV (42) and caregivers (10)	Perinatal	15–19
Mutumba, et al. [44]	Qualitative cross sectional	Uganda	ALHIV (38)	Perinatal	13–19
Mackworth‐Young, et al. [45]	Qualitative cross sectional	Zambia	ALHIV (24)	Perinatal and behavioural	15–18
Petersen, et al. [46]	Qualitative cross sectional	South Africa	ALHIV (25) and caregivers (15)	NR	14–16
Ramaiya, et al. [47]	Qualitative cross sectional	Tanzania	ALHIV (62)	NR	13–23
Kubanji, et al. [48]	Qualitative cross sectional	Botswana	ALHIV (26), caregivers (8) and HCP (25)	NR	15–19
Crowley, et al. [49]	Qualitative cross sectional	South Africa	ALHIV (44), caregivers (6) and HCP (6)	NR	13–18
Daniel [50]	Qualitative longitudinal	Tanzania	ALHIV (13) and HCP (4)	Perinatal	10–15

Abbreviations: ALHIV, adolescent living with HIV; HCPs, healthcare providers; MoH, Ministry of Health; NR, not reported.

### 2.4 Data analysis and synthesis

The review used a combined analysis approach; grounded theory, generating sub‐themes from the free codes that were captured in the text of the articles, and a thematic analysis approach was used to analyse and synthesize the codes into themes. In NViVo, reported needs were exhaustively coded line by line to ensure a thorough representation of the information from adolescents. The analysis process was iterative as themes were modified or generated following multiple reads of the articles and deductive reasoning of the meanings. Although these articles already had inherent interpretations from the authors, further interpretation of the codes through a combination of a realist assessment and constructionist approach [21] was used for a more in‐depth and balanced assessment of the findings. A priority ranking was created from studies that included multiple health‐related needs. Meta‐analysis was not possible due to the range of outcomes and methods expected in the literature.

## 3 RESULTS AND DISCUSSION

### 3.1 Eligible studies

**Figure 1:**
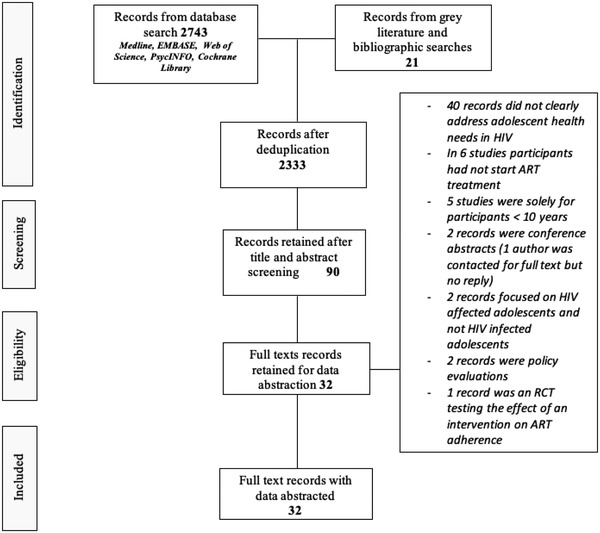
Review flow chart, illustrating the PRISMA study selection process.

The searches generated 2764 records, of which 431 were duplicates, yielding 2333 records without duplicates. The titles and abstracts of deduplicated records were screened, and 90 records were retained for full text read. Of the 90, 58 records were not eligible for the following reasons; did not address the health needs of ALHIV (*n* = 40), did not clearly demonstrate that ALHIV were on ART (*n* = 6), studies solely of children <10 years (*n* = 5), conference abstracts for which the review team contacted the authors for full texts and received no feedback (*n* = 2), studies focused on adolescents affected by but not living with HIV (*n* = 2), policy evaluations (*n* = 2) and a trial testing the effect of an intervention without clear reference to the health needs of ALHIV (*n* = 1). A total of 32 studies were retained for further review (Figure 1).

### 3.2 Study characteristics

**Figure 2:**
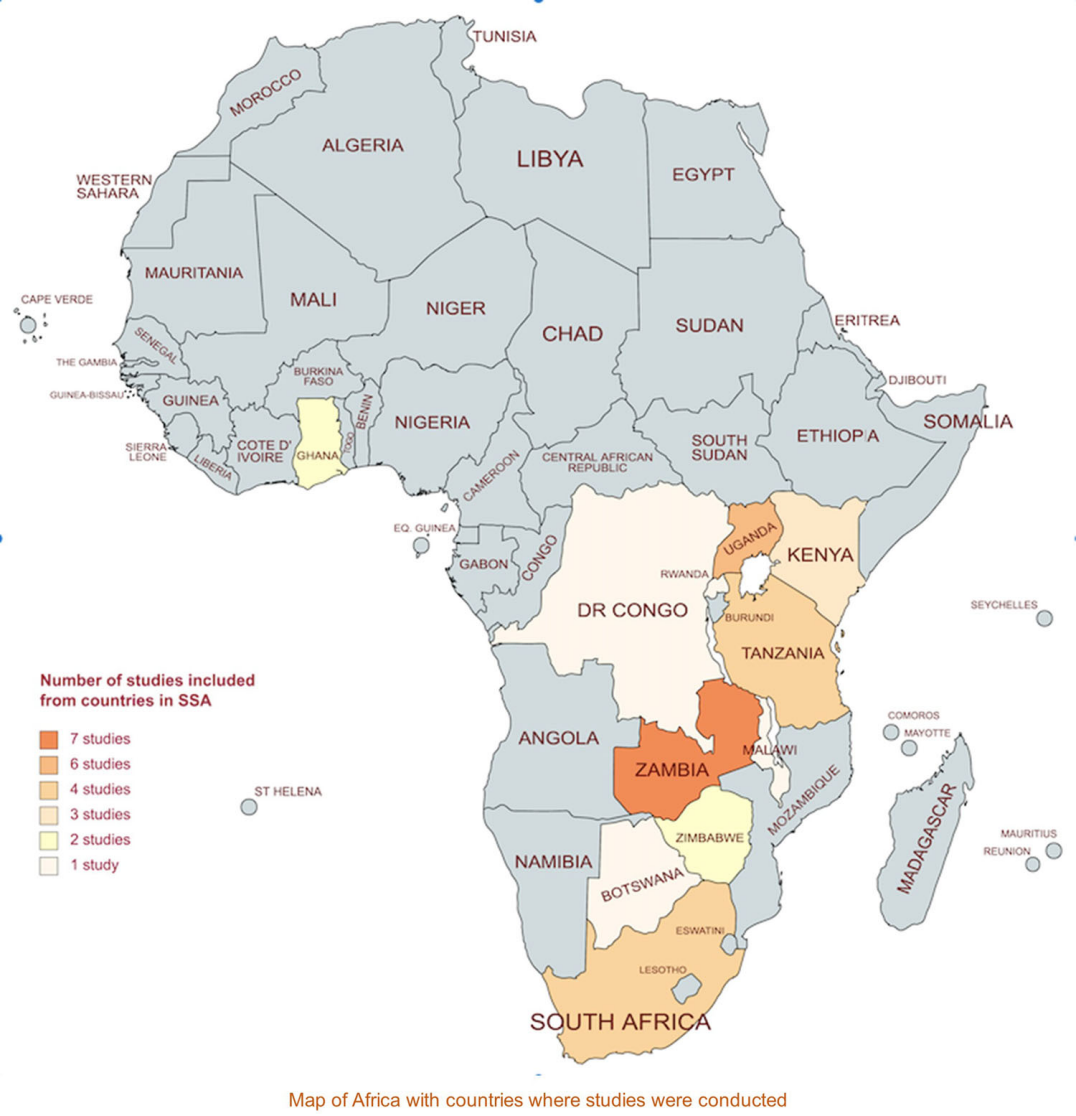
Countries where studies were conducted.

The 32 studies retained were published between 2008 and 2019. The age range of study participants in all 32 studies was 8–24 years, but only data for those aged 10–19 years were abstracted. Of the 32 eligible studies, 26 were qualitative studies, five were mixed methods and one was a quantitative survey. Thirty studies presented the health‐related needs from the perspectives of ALHIV, 18 included the perspective of caregivers, 13 included the perspective of healthcare providers, two investigated the perspective of HIV program managers, representatives of the Ministry of Health and HIV partners, and one study included school teachers and HIV‐negative adolescents (Table 1). Ten studies reported the health‐related needs of adolescents with perinatally acquired HIV, five from both perinatally and horizontally acquired ALHIV, and 17 studies did not specify the mode of acquisition. The included studies were conducted in Zambia (*n* = 7), Uganda (*n* = 6), Tanzania (*n* = 4), South Africa (*n* = 4), Kenya (*n* = 3), Ghana (*n* = 2), Zimbabwe (*n* = 2), Rwanda (*n* = 1), Malawi (*n* = 1), Botswana (*n* = 1) and Democratic Republic of Congo (*n* = 1) (Table 1 and Figure 2).

### 3.3 Health‐related needs of ALHIV on ART

Seven categories of health‐related needs among ALHIV were identified in the following descending order of occurrence: psychosocial needs; dependency of care; self‐management needs; non‐responsive health services; need for food, financial and material support; inadequate information about HIV; and developmental and growth needs (Table 2).

**Table 2:** Compendium of health‐related needs among ALHIV on ART in SSA, according to participants in different social ecological levels

Social ecological level	Priority themes for health‐related needs among ALHIV (from most to least important)	Sub‐theme (needs)
Adolescents living with HIV	Psychosocial needs	Stigma
		Disclosure difficulties
		Difficulty in accepting HIV diagnosis
		Community and social support
		Secrecy during ART
		Communication challenges around HIV
		Violence (physical, domestic and sexual)
	Self‐management and medication adherence needs	Difficulty in identifying and developing better coping strategies against HIV and ART
		Need for medication adherence support
		Difficulty in tolerating ART regimen (pill taste, size, number and frequency) and side effects
	Dependency during care	Need for family support
		Need for healthcare provider support
		Lack of adolescent autonomy during treatment
		Self‐care and responsibilities for others
	Non‐responsive health services	Non‐adolescent friendly services
		School system not considerate of ALHIV needs
		Negative health provider attitudes towards ALHIV when they miss ARVs
		High facility turnaround time during ARV refills
		Desire for more information about SRH
		Mental health needs to overcome HIV‐induced depression and stress
	Inadequate information about HIV and related issues	Desire for more knowledge about HIV
		Misinformation, misconception and misperceptions about HIV and related events
		Sexual and reproductive health knowledge (condom use and contraceptive use), HIV transmission
	Financial challenges	Financial, food and material need
	Developmental and growth challenges	Desire to experience sex
		Desire to experience parenthood
		Desire to love and be loved in return
Caregivers	Psychosocial needs	Stigma
		Difficulty in accepting HIV diagnosis
		Reliance on spiritual and religious support
		Disclosure difficulties
		Desire to maintain secrecy during ART
	Financial challenges	Financial, food and material need
		Cost of transportation to healthcare facilities
	Inadequate information about HIV and related issues	Inadequate information about HIV and related issues
		Deception from carers to ALHIV about the realities of HIV
	Medication adherence needs	Need for medication adherence support
	Dependency during care	Inadequate family support
		Negative healthcare provider attitude towards ALHIV
		Effect of HIV on ALHIV and caregiver health
		Lack of adolescent autonomy
	Non‐responsive services	Non‐responsive school systems on HIV and related issues, negative attitudes of healthcare personnel towards ALHIV
		Mental health needs to overcome HIV‐induced depression and stress
Healthcare providers	Non‐responsive health services	No adolescent‐friendly services
		Lack of skills by healthcare providers to care for ALHIV
		Need for sexual and reproductive care for ALHIV
		Impact of school on health services for ALHIV
		ART stock‐out
		Mental health needs to overcome HIV‐induced depression and stress
	Dependency during care	Need for family support
		Desire for healthcare provider support
		Lack of adolescent autonomy
		Poor physical and mental health for ALHIV
	Psychosocial needs	Disclosure difficulty
		Need for smooth communication between ALHIV and healthcare providers
		Community and peer support
		Desire to maintain secrecy
		Stigma
	Inadequate information about HIV and other related conditions	Need for ALHIV to understand HIV and related issues
		Sexual and reproductive health knowledge (condom use and contraceptive use), HIV transmission
	Medication adherence needs	Medication adherence difficulties
	Financial challenges	Financial, food and material need
		Cost of transportation to health facilities

#### 3.3.1 Psychosocial needs

Twenty‐nine studies reported the psychosocial challenges ALHIV encounter while taking ART and their day‐to‐day experiences of living with HIV. Stigma was the most reported psychosocial challenge, and mostly focused on the school environment, leading to poor ART adherence and school dropout. The studies also revealed the challenges ALHIV encounter in disclosing their HIV status, difficulties accepting a positive HIV diagnosis (especially for perinatally acquired ALHIV), the need to keep HIV medications hidden from people outside their network and situations in which ALHIV experienced physical and sexual violence.

#### 3.3.2 Dependency of care

A total of 23 studies described ALHIV's expressed and felt need around dependency during care, identifying family, community, social and healthcare provider support as crucial. ALHIV also described their general lack of autonomy to make decisions about their care and ART medication. Two studies described circumstances where ALHIV became autonomous because they did not have a guardian, taking decisions for themselves and caring for other household members, such as their siblings and elderly caregivers. Caregivers also highlighted the importance of spiritual and religious support in adolescents’ care and adherence to medication.

#### 3.3.3 Self‐management needs

Self‐management of ART among ALHIV was highlighted as a key challenge by all participants. Twenty studies reported circumstances in which ALHIV had difficulty identifying and developing coping strategies for managing their ART, which led to their desire for medication adherence support. The studies also highlighted that ALHIV desire better strategies to cope with everyday ART and their side effects, and more palatable ART regimens.

#### 3.3.4 Non‐responsive health services

Adolescent‐friendly services were identified as an unmet need by ALHIV, caregivers and healthcare providers in 17 studies. The specific issues that highlighted this need include lack of “adolescent only” spaces in HIV treatment facilities, long waiting times for ART refills, perceived negative (reprimanding) attitudes of some healthcare providers towards ALHIV when they miss doses and stock‐outs of ART. Healthcare providers and ALHIV also expressed the need for sexual and reproductive health (SRH) services tailored to the needs of ALHIV on ART, and the organization of health services that do not conflict with adolescent school schedules. Four studies identified the importance of mental health services for ALHIV on ART who often suffered from depression and stress, partly because of the stigma and discrimination they experience in society.

#### 3.3.5 Inadequate information about HIV

Fifteen studies highlighted the necessity for ALHIV to receive adequate knowledge about HIV and ART to fight misinformation, misconception and misperceptions.

#### 3.3.6 Financial challenges

In 13 studies, ALHIV, caregivers and healthcare providers expressed the need for food, financial and material support for ALHIV receiving ART. Financial support was directed towards catering for food and transportation cost to health facilities for ART refills.

#### 3.3.7 Developmental and growth needs

In eight studies, ALHIV expressed the desire to explore their sexuality and engage in intimate relationships. In four studies, ALHIV described the desire to have children.

### 3.4 Prioritization of needs by socio‐ecological level

ALHIV, their caregivers and healthcare providers had different perceptions of the priority needs of ALHIV on ART (Table 3). ALHIV focused on psychosocial issues (stigma, difficulties in disclosing status to people outside the HIV care network, challenges in maintaining privacy during ART medication, difficulties in accepting an HIV‐positive diagnosis and reports of HIV‐associated violence at home, school and within the community). Addressing violence was cited by ALHIV as the most challenging unmet need for ALHIV in most studies. The caregivers acknowledged the importance of psychosocial challenges expressed by ALHIV, but focused more on the need for food, financial and material support as key to the wellbeing of ALHIV. Healthcare providers focused on the non‐responsive nature of the health services as the main unmet health need for ALHIV on ART. They highlighted the lack of policies to enable effective planning and implementation of adolescent‐friendly activities within the healthcare facilities, the need for integration of services for ALHIV and inadequate logistic systems for ART supplies [28].

**Table 3:** Order of prioritization of health‐related needs for ALHIV on ART according to participants in different social ecological levels (from most to least important)

Adolescents	Caregivers	Healthcare providers
Psychosocial needs	Psychosocial needs	Non‐responsive health systems
Self‐management and medication adherence needs	Financial, food and material needs	Dependency of care
Dependency of care	Inadequate information about on HIV and ART	Psychosocial needs
Non‐responsive health systems	Self‐management and medication adherence needs	Inadequate information about HIV, ART and related services
Inadequate information about HIV, ART and related services	Dependency of care	Self‐management and medication adherence needs
Financial, food and material needs	Non‐responsive health services	Financial, food and material needs
Developmental and growth challenges		

This review is the first to systematically synthesize the reported unmet health‐related needs of ALHIV on ART in SSA, highlighting possible areas for intervention in adolescent ART care. The results yielded seven broad categories of health‐related needs that ALHIV experience while on ART: psychosocial needs; dependency of care; self‐management needs; non‐responsive health services; need for food, financial and material support; inadequate information about HIV; and developmental and growth needs. There were different perspectives of the priority health‐related needs for ALHIV when described by ALHIV, caregivers and healthcare providers.

### 3.5 Prioritization of needs at different socio‐ecological levels

#### 3.5.1 Perspectives of ALHIV

This review highlighted the divergent opinions in prioritization of needs between the different stakeholders involved in adolescent HIV treatment and care. Psychosocial needs (stigma and disclosure) dominated the health‐related needs expressed by ALHIV on ART and their caregivers. ALHIV struggle with stigma, including internalized stigma, provider‐based stigma and public stigma mostly experienced within school settings [51, 52]. Schools are key social environments where most adolescents spend their time, yet ALHIV continue to report complex experiences within the classroom environment, including stigma from peers and teachers. This is mostly driven by teachers and the nature of the message they convey about HIV [52]. However, only two studies have been conducted in SSA on interventions to improve the knowledge, attitude and behaviour of teachers, and few school‐based stigma‐reduction interventions for children and adolescents [52, 53]. More school‐based interventions are needed to reduce stigma towards people living with HIV as these environments are fundamental to the wellbeing of ALHIV, their ART adherence outcomes and school retention rates [54]. ALHIV may struggle with increased self‐stigma through predominantly ART adherence intervention‐focused approaches [7, 8, 55]. A stigma reduction approach with a more general public focus may be more effective.

Our review identified mental health services as an important unmet need for ALHIV. Though adolescents may experience mental health issues, such as depression and stress, due to a variety of causes, most ALHIV attributed them to the protracted effect of stigma from the society. A review of mental health challenges of ALHIV reported the critical lack of efforts to measure the impact of mental health challenges on ALHIV and to refine health systems in LMICS to be responsive to the mental health needs of ALHIV [56, 57].

This review identified a key unmet need related to medication adherence. ALHIV reported difficulty in identifying and developing better coping strategies for ART; difficulties in tolerating ART regimen and side effects; and the need for medication adherence support. Difficulties in coping with therapy and poor adherence is a universal challenge for those growing up with chronic diseases in childhood [58]. Self‐management of HIV and ART among ALHIV requires knowledge about HIV and ART empowers them to prioritize specific aspects of their care to achieve better treatment outcomes [49]. Self‐management also relies on the network of support ALHIV receive from immediate family, healthcare personnel and peers. However, a recent review on self‐management interventions did not find evidence on the effectiveness of self‐management on ART outcome among ALHIV [59]. Increased investment in self‐care interventions that promote social spaces for ALHIV, peer‐to‐peer support activities and development of individualized ART adherence plans can improve ALHIV's agency during ART medication [60, 61]. In addition, the modification of ART regimens for ALHIV may improve adherence and self‐management [58]. These include single tablet/day regimens, use of ART with less side effects and reduced chances of regimen switch, such as dolutegravir, and the growing prospects of long‐acting ART (under clinical trial).

Accurate information about HIV and related issues that affect the wellbeing of ALHIV is critical in their successful management of HIV. Our review highlights the need for increased education on HIV, including addressing myths and misperceptions, SRH services, including condom use, contraception and STIs, clarifying HIV transmission and increasing the knowledge on prevention of HIV from mother to child. This aligns with other studies, which have further identified ALHIV who acquired HIV from horizontal transmission to be more knowledgeable than those with perinatally acquired HIV [62]. Chory and colleagues substantively elaborated on this need using a WhatsApp mobile intervention, which provided ALHIV with a private and safe platform to express their needs and concerns during HIV treatment. However, the feasibility of rolling out this digital approach remains questionable as it risks creating a digital canyon between ALHIV who can access the internet and those who cannot.

Eight studies in our review reported developmental and growth needs, where ALHIV expressed the desire to explore their sexuality, were anxious about parenthood and engaging in intimate relationships. Negotiating sexuality and sexual debut has been a major challenge, especially for perinatally infected ALHIV who are living with HIV before sexual debut and may not understand the limits of how much they are permitted to explore in terms of sexuality compared to their peers [45, 58]. Caregivers and healthcare providers did not perceive this as a need in this review. This is not unexpected as caregivers and healthcare providers are more concerned about the repercussions of sexual activity on treatment outcomes among ALHIV [11, 63]. In addition, sexuality education remains taboo in many societies in SSA. A comprehensive sexuality education approach that integrates ALHIV, parents, community members, teachers and key decision makers during the design of interventions may be more effective [64].

#### 3.5.2 Perspectives of caregivers

Caregivers often cited the economic and material challenges associated with treatment and care for ALHIV. This is not unexpected as caregivers bear the brunt of the responsibility to care and support ALHIV with food, material, financial, emotional, psychological and social support. Food is critical because ALHIV are expected to eat well to minimize the side effects of ART [22, 36]. Caregivers in SSA may be unemployed, HIV positive, sick and unable to work, may be challenged with food insecurity for various reasons or may have other competing financial commitments [32, 36]. This contradicts the report by Wiener and colleagues, where parents of children living with HIV prioritized mental health and health education of ALHIV [65]. This was likely because the study was conducted in the United States (high‐income setting) and in the 1990s when knowledge about HIV was still limited. Cash support interventions for ALHIV and caregivers should complement interventions to improve treatment outcomes among ALHIV [66]. Interventions addressing economic insecurity demonstrate the potential to bolster ART adherence and health outcomes among ALHIV in low‐resource settings [67].

#### 3.5.3 Perspectives of healthcare providers

**Figure 3:**
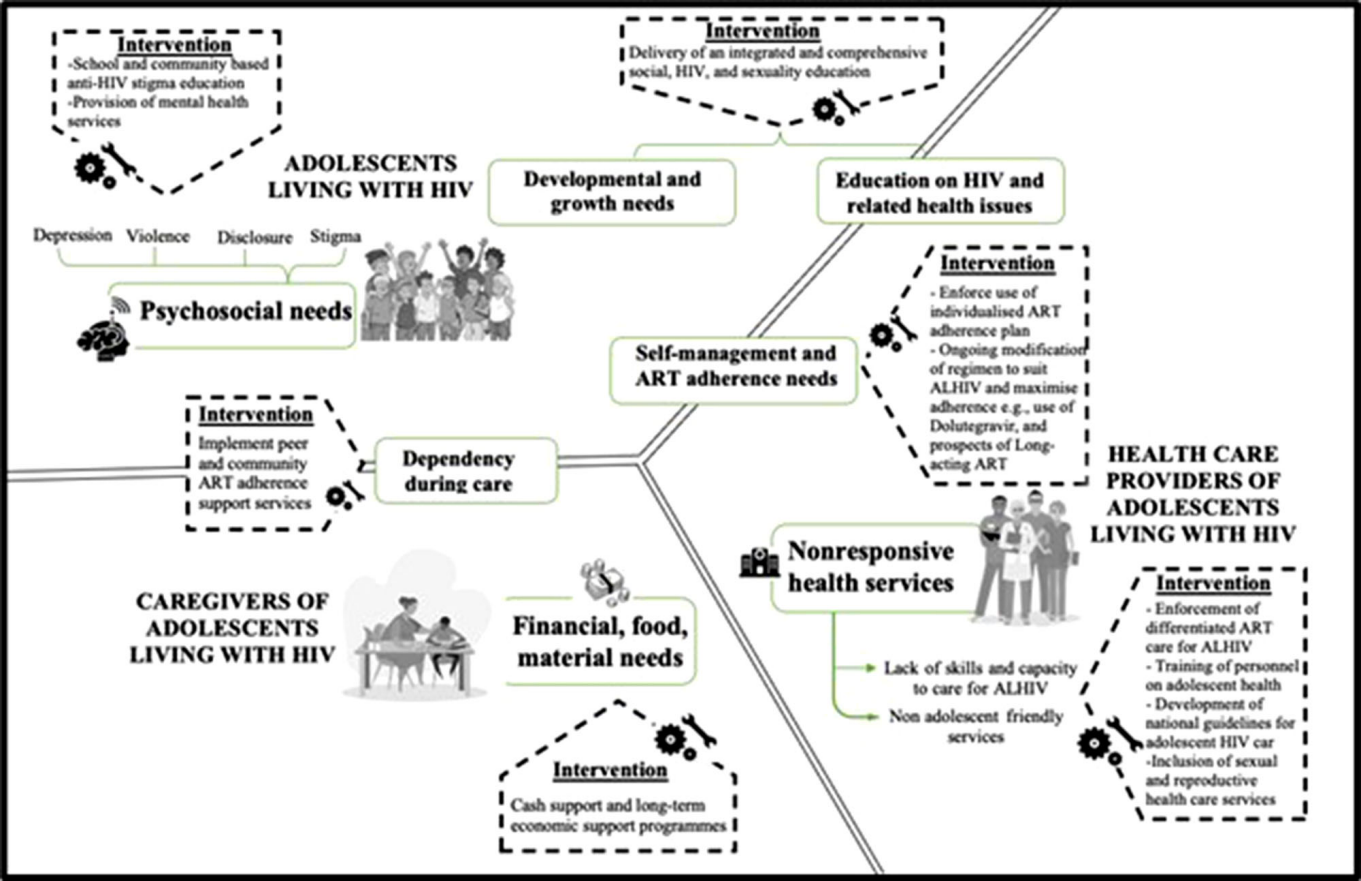
Summary of health‐related needs of ALHIV on ART, and examples of interventions that can be implemented to address the needs.

Healthcare providers were open about their lack of capacity to provide adolescent‐friendly services, which were either non‐existent or poorly implemented in HIV treatment facilities, as well as the non‐complementarity between school programs and adolescent HIV treatment programs. Lack of skilled staff to manage ALHIV and weak implementation of adolescent‐friendly services is a leading challenge in structural efforts to improve holistic adolescent HIV care services in LMICs [68, 69]. The need to integrate the school needs of ALHIV into their treatment programs cannot be overemphasized. It is important to promote differentiated service delivery care for ALHIV with multi‐month prescription of ART for treatment stable ALHIV, individualized treatment plans for unstable patients, taking into consideration their school schedules [68, 69]. Some countries in SSA lack national guidelines on treatment and care for ALHIV. Designing adolescent‐friendly national HIV treatment and care guidelines and ensuring their implementation and effective use will improve the access and quality of adolescent‐friendly services. Incorporating guidelines on adolescent‐friendly service into the training curriculum for healthcare providers and counsellors will improve the skills of healthcare providers (Figure 3).

This is a review of the evidence on felt and expressed health‐related needs of ALHIV on ART, and it only included papers in which the nature of the needs was defined by the ALHIV or their caregivers. A limitation of the review is that research on pre‐defined or normative needs was not included. Another limitation of the review is that the opinions of adolescent HIV program managers, civil society agents, policy makers and other HIV partners were not captured. Including them may have identified the structural challenges that affect the services provided to ALHIV and what to them constitutes the most important health‐related needs for ALHIV receiving ART. Additional research to investigate the perspectives of decision and policy makers on the needs of ALHIV on ART should be conducted to complement the findings of this review and provide a more holistic picture of the health‐related needs of ALHIV on ART according to all the levels of the socio‐ecological model for a more comprehensive and informed public health response. The findings of this review are informative on the self‐perceived and perceived needs of ALHIV, but other forms of research may identify needs that are not those perceived by ALHIV, caregivers or service providers. Mode of HIV acquisition was not reported for 17/32 studies.

Finally, studies included in this review were conducted prior to the COVID‐19 pandemic. It is possible that the current needs of ALHIV on ART may have been moderated by their experiences during the COVID‐19 pandemic. Research on the effects of the COVID‐19 pandemic on the health‐related needs of ALHIV on ART will shed more light on the challenges ALHIV on ART experience during the pandemic.

## 4 CONCLUSIONS

ART adherence remains a major challenge among ALHIV, and understanding their health‐related needs is critical for effective programming. In this review, we demonstrate the health‐related needs of ALHIV on ART in SSA as identified by ALHIV, caregivers and healthcare providers, and highlighted their implications for care and treatment outcomes. To respond effectively to the health needs of ALHIV and improve their ART adherence, key areas of focus for interventions include stigma reduction, especially in school environments, supporting ALHIV with overcoming challenges relating to disclosure, as well as innovative coping mechanisms for ART. Interventions that address the health needs of ALHIV from the perspective of caregivers and healthcare providers, such as financial support schemes and adolescent‐friendly healthcare strategies respectively, should supplement efforts to improve adolescent ART adherence outcomes.

## AUTHORS’ CONTRIBUTIONS

EDC: Conceptualization, literature search, records screening, data extraction, analysis, writing the initial draft, reviewing and editing. AF: records screening, data extraction, reviewing and editing. JS, HAW and VS: Conceptualization, reviewing and editing.

## FUNDING

This review was funded by the Commonwealth Scholarship awarded to EDC (Open Dreams Cameroon/HALI‐Commonwealth Scholar) as part of his PhD project. HAW and VS received funding from the UK Medical Research Council (MRC) and the UK Foreign, Commonwealth and Development Office (FCDO) under the MRC/FCDO Concordat agreement which is also part of the EDCTP2 programme supported by the European Union (grant number MR/R010161/1).

## Supporting information


**File S1**: Search strategy.
This illustrates the search terms and strategy that was used in MEDLINE.

## Data Availability

The authors declare no competing interests.
