# Supplementary material for: Health‐related needs reported by adolescents living with HIV and receiving antiretroviral therapy in sub‐Saharan Africa: a systematic literature review
Source: J Int AIDS Soc. 2022 Aug 19;25(8):e25921. doi: 10.1002/jia2.25921 (PMC9389275; doi:10.1002/jia2.25921)
Supplement: Supplementary file 1 — File S1: Search strategy. This illustrates the search terms and strategy that was used in MEDLINE. [file JIA2-25-e25921-s001.docx]

**Additional file 1**

Search strategy in MEDLINE

MEDLINE

| Search no | Search term | Number of articles |
| --- | --- | --- |
| 1 | Needs OR Health needs OR health desire OR health wants | 317399 |
| 2 | MeSH: (["Health Services Needs and Demand"](https://ovidsp.dc1.ovid.com/sp-4.04.0a/ovidweb.cgi?&Controlled+Vocabulary=Mapping%7c2&Return=mapping&S=GDMLFPJDMHACEAAJKPCKIHGJEFPIAA00)) | 52135 |
| 3 | 1 OR 2 | 317399 |
| 4 | Adolescen* OR youth* OR teen* OR young adult OR juvenile | 2463976 |
| 5 | MeSH: ([Adolescent Behavior](https://ovidsp.dc1.ovid.com/sp-4.04.0a/ovidweb.cgi?&Controlled+Vocabulary=Mapping%7c1&Return=mapping&S=EPFKFPDOMCACFAHEKPCKGGDKFMLHAA00) OR [Adolescent Health](https://ovidsp.dc1.ovid.com/sp-4.04.0a/ovidweb.cgi?&Controlled+Vocabulary=Mapping%7c3&Return=mapping&S=EPFKFPDOMCACFAHEKPCKGGDKFMLHAA00) OR [Adolescent Health Services](https://ovidsp.dc1.ovid.com/sp-4.04.0a/ovidweb.cgi?&Controlled+Vocabulary=Mapping%7c4&Return=mapping&S=EPFKFPDOMCACFAHEKPCKGGDKFMLHAA00)) | 1987116 |
| 6 | 4 OR 5 | 2463976 |
| 7 | (HIV OR hiv1 OR hiv2 OR hiv infect* OR human immunodeficiency virus OR human immune deficiency virus OR ((human immun*) AND (deficiency virus)) OR acquired immunodeficiency syndromes OR acquired immune deficiency syndrome OR acquired immunodeficiency syndrome OR acquired immunedeficiency syndrome OR ((acquired immun*) AND (deficiency syndrome)) OR HIV OR AIDS) | 421880 |
| 8 | MeSH: HIV infections | 19509 |
| 9 | 7 OR 8 | 421880 |
| 10 | Antiretroviral OR Antiretroviral therapy OR Anti-retroviral OR Anti-retroviral therapy OR ART OR ARV OR Antiretroviral medication OR Antiretroviral regimen OR Antiretroviral administration OR Highly active antiretroviral OR (MH “Art”) OR (MH “Art Therapy”) | 128819 |
| 11 | Antiretroviral Therapy, Highly Active/ or Anti-HIV Agents/ | 56606 |
| 12 | 10 OR 11 | 149266 |
| 13 | Sub-Saharan Africa OR Africa South of Sahara OR Saharan Africa OR Africa OR Angola OR Benin OR Botswana OR Burkina Faso OR Burundi OR Cameroon OR Cape Verde OR Central African Republic OR Chad OR Comoros OR Congo OR Democratic Republic of Congo OR Ivory Coast OR Djibouti OR Equatorial Guinea OR Eritrea OR Ethiopia OR Gabon OR The Gambia OR Ghana OR Guinea OR Guinea-Bissau OR Kenya OR Lesotho OR Liberia OR Madagascar OR Malawi OR Mali OR Mauritania OR Mauritius OR Mozambique OR Namibia OR Niger OR Nigeria OR Reunion Island OR Rwanda OR Sao Tome and Principe OR Senegal OR Seychelles OR Sierra Leone OR Somalia OR South Africa OR Sudan OR Swaziland OR Tanzania OR Togo OR Uganda OR Western Sahara OR Zambia OR Zimbabwe |  |
| 14 | 3 AND 6 AND 9 AND 12 AND 13 | 2892 |
